# A comprehensive review of the SLMTA literature part 1: Content analysis and future priorities

**DOI:** 10.4102/ajlm.v3i2.265

**Published:** 2014-11-03

**Authors:** Elizabeth T. Luman, Katy Yao, John N. Nkengasong

**Affiliations:** 1 International Laboratory Branch, Division of Global HIV/AIDS, Center for Global Health, US Centers for Disease Control and Prevention, Atlanta, Georgia, United States

## Abstract

**Background:**

Since its introduction in 2009, the Strengthening Laboratory Management Toward Accreditation (SLMTA) programme has been implemented widely throughout Africa, as well as in the Caribbean, Central and South America, and Southeast Asia.

**Objective:**

We compiled results from local, national and global studies to provide a broad view of the programme and identify directions for the future. The review consists of two companion papers; this paper focuses on content analysis, examining various thematic components of the SLMTA programme and future priorities.

**Methods:**

A systematic literature search identified 28 published articles about implementing the SLMTA programme. Results for various components of the SLMTA programme were reviewed and summarised.

**Results:**

Local and national studies provide substantial information on previous experiences with quality management systems; variations on SLMTA implementation; building human resource capacity for trainers, mentors and auditors; the benefits and effectiveness of various types of mentorship; the importance of management buy-in to ensure country ownership; the need to instill a culture of quality in the laboratory; success factors and challenges; and future directions for the programme.

**Conclusions:**

Local, national and global results suggest that the SLMTA programme has been overwhelmingly successful in transforming laboratory quality management. There is an urgent need to move forward in four strategic directions: progression (continued improvement in SLMTA laboratories), saturation (additional laboratories within countries that have implemented SLMTA), expansion (implementation in additional countries), and extension (adapting SLMTA for implementation beyond the laboratory), to lead to transformation of overall health systems and patient care.

## Introduction

The Strengthening Laboratory Management Toward Accreditation (SLMTA) programme is a structured quality improvement curriculum designed to achieve immediate and tangible advances in health service delivery.^1^ The central feature of this intervention is its emphasis on the ‘how-to’ of implementing quality management systems (QMS) by translating the ‘what’ (concepts, principles, theories, guidelines and standards) into practical behaviours, laboratory practices and daily routines, through hands-on practice during training and improvement projects in trainees’ home laboratories. The importance of practical training dedicated to building management capacity has been well deliberated in a series of working papers published by the World Health Organization (WHO) on strengthening leadership and management in low-income countries, titled ‘Making Health Systems Work’.^2,3,4,5^

In the five years since its introduction in 2009, the SLMTA programme has been implemented widely throughout the developing world.^1^ In December 2012, SLMTA country coordinators and implementers gathered at a two-day symposium in Cape Town, South Africa to discuss their experiences and lessons learned. It was evident that a substantial amount of programmatic expertise had been gained. A supplemental issue of the *African Journal of Laboratory Medicine* (AJLM) was commissioned in order to document and share successes and challenges, summarize laboratory- and country-specific analyses, and publish global programme data.

This systematic literature review aims to compile existing fragmented results into a comprehensive report, to provide a broad view of the programme and to identify directions for the future. Because of the large volume of information collected, the review has been published in two parts. Part 1 focuses on content analysis, examining various thematic components of the SLMTA programme and future priorities. Part 2, published separately, compiles the quantitative data reported in the publications, examines scores and indicators, and uses meta-analysis to augment the results.^6^

## Research methods and design

A comprehensive search of electronic bibliographic databases was performed, including Medline and the Directory of Open Access Journals, using the keyword ‘SLMTA’. SLMTA country programme leaders and partner agencies were contacted so as to identify additional sources. We included all published and in-press studies that discussed the SLMTA programme. The majority of the search results were in-press manuscripts being prepared for the supplemental issue of AJLM focusing on the SLMTA programme, called ‘Transforming the Quality of Laboratory Medicine through the Strengthening Laboratory Management Toward Accreditation Program’; authors of this review coordinated the issue.

## Results and discussion

### Literature search results

We identified 28 published manuscripts focusing on the SLMTA programme,^1,7–33^ including 26 published concurrently with this article,^1,7-20,22-31,33^ one previously-published article summarising the development and methodology of the SLMTA programme^32^ and one previously-published study regarding SLMTA implementation in Lesotho^21^ (Table 1). Six papers presented experiences from a single laboratory^7,10,17,26,28^ or a single hospital,^9^ 14 presented data from two to 45 laboratories within a single African country,^8, 12,–15,18,–22,25,29–31^ one discussed activities in two Southeast Asian countries,^23^ one covered data from four countries in the Caribbean Region^11^ and six had a general or global focus.^1,16,23,24,32,33^ In total, these studies included detailed information on SLMTA implementation in 211 laboratories in 18 countries.

**TABLE 1:** Characteristics of published SLMTA studies.

Study	Country/Countries	Level of study	Number of laboratories	Years of study
Andiric et al.^7^	Tanzania	Select laboratory	1	2010–2011
Audu et al.^8^	Nigeria	Select laboratories	2	2010–2013
Eno et al.^9^	Cameroon	Select hospital	1	2011–2012
Gachuki et al.^10^	Kenya	Select laboratory	1	2010–2013
Guevaraet al.^11^	Bahamas, Jamaica, Barbados, Trinidad and Tobago	One cohort	5	2011–2013
Hiwotu et al.^12^	Ethiopia	Two cohorts	45	2010–2012
Lulie et al.^13^	Ethiopia	Select laboratories	17	2013
Maina et al.^14^	Kenya	Select laboratories	5	2011–2012
Makokha et al.^15^	Kenya	Select laboratories	8	2010–2011
Maruta et al.^16^	NA	Global	NA	2009–2013
Maruti et al.^17^	Kenya	Select laboratory	1	2011–2013
Masamha et al.^18^	Mozambique	One cohort	8	2010–2012
Mataranyika et al.^19^	Namibia	One cohort	6	2012–2013
Mokobela et al.^20^	Bostwana	One cohort	7	2010–2011
Mothabeng et al.^21^	Lesotho	Two cohorts	18	2010–2011
Ndasi et al.^22^	Cameroon	One cohort	5	2009–2012
Nguyen et al.^23^	Vietnam and Cambodia	General	NA	2012–2013
Nkengasong et al.^24^	NA	General	NA	NA
Nkrumah et al.^25^	Ghana	Three cohorts	15	2011–2013
Nkwawir et al.^26^	Cameroon	Select laboratory	1	2009–2013
Noble et al.^27^	NA	General	NA	NA
Ntshambiwa et al.^28^	Bostwana	Select laboratory	1	2010–2013
Nzabahimana et al.^29^	Rwanda	Three cohorts	15	2010–2013
Nzombe et al.^30^	Zimbabwe	One cohort	19	2010–2012
Shumba et al.^31^	Zimbabwe	Two cohorts	30	2010–2012
Yao et al.^32^	NA	General	NA	NA
Yao et al.^1^	NA	General	NA	2009–2013
Yao et al.^33^	47 countries*	Global	617	2010–2013

SLMTA, Strengthening Laboratory Management Toward Accreditation; NA, not applicable.

* Angola, Antigua, Bahamas, Barbados, Belize, Botswana, Burundi, Cambodia, Cameroon, Columbia, Costa Rica, Cote d’Ivoire, Democratic Republic of the Congo, Dominica, Dominican Republic, El Salvador, Ethiopia, Ghana, Grenada, Guatemala, Haiti, Honduras, Jamaica, Kenya, Lesotho, Malawi, Mozambique, Namibia, Nicaragua, Nigeria, Panama, Peru, Rwanda, Sierra Leone, South Africa, South Sudan, Saint Kitts, Saint Lucia, Saint Vincent, Suriname, Swaziland, Tanzania, Trinidad and Tobago, Uganda, Vietnam, Zambia, Zimbabwe.

### Previous experience with quality management systems

A recent study summarised the scarcity of accreditation in public laboratories throughout sub-Saharan Africa.^34^ However, the results from the SLMTA laboratories paint an even more problematic picture – not only are laboratories in developing countries not meeting the international quality standards needed for accreditation, but the level of quality management is extremely low. Several authors reported that their laboratories had no prior experience with QMS,^7,10,17,22^ with one study reporting that prior to SLMTA, ‘the idea of QMS was entirely new to most laboratory staff in the selected facilities, where a culture of quality has been lacking’.^22^ The low level of baseline audit scores across the programme – 84% of laboratories did not reach even one star on the WHO Regional Office for Africa’s (AFRO) Stepwise Laboratory Quality Improvement Process Towards Accreditation (SLIPTA) five-star scale and the mean baseline audit score was 39% – seems to confirm this assessment.^33^ These findings are supported by a study in Kampala, Uganda, which found that only 5% of audited laboratories in the city had reached one star on the SLIPTA quality scale.^35^

Previous experience in establishing QMS was limited in these countries and what had been done was largely reported to be ineffective. One study of SLMTA implementation in the Caribbean Region reported that ‘past laboratory assessments and training did not provide them with a structured roadmap to assist in implementation; as a result, the majority of these laboratories did not initiate the process of QMS development and implementation’.^11^ Another reported that ‘prior to the introduction of SLMTA, several trainings and quality improvement initiatives had been implemented in hospitals and laboratories in Ethiopia, but little improvement was noted’.^12^ In Botswana, there had been ‘slow progress in implementing QMS’, and authors noted that ‘previous training of healthcare workers has focused on general management topics rather than identifying tangible tasks to bring about change, making the training difficult to apply in the laboratory’.^28^

### The drive for action

Several authors report that implementation of SLMTA came after years of neglect of laboratories. In many cases, these circumstances improved with the advent of the US President’s Emergency Plan for AIDS Relief (PEPFAR) in 2003,^7,11,14,15,22,27^ which emphasised the importance of quality laboratory tests and infused much-needed capital into the laboratory systems.^36^ Other international programmes also played a key role, such as the Global Fund to Fight AIDS, Tuberculosis and Malaria^37^ and the Global Health Initiative.^38^ Concurrently, several regional and global policy statements called for strengthening public medical laboratories: the Maputo Declaration, which called on ‘national governments to support laboratory systems as a priority’;^39^ the Lyon Statement on the need for laboratory QMS and accreditation of National Reference Laboratories;^40^ and the Yaoundé Resolution, in which WHO AFRO recognised the need to further strengthen public health laboratories and to encourage member states ‘to develop or strengthen comprehensive national laboratory policies’.^41^

Authors noted that also during this timeframe, countries began to develop five-year laboratory strategic plans – Kenya^14,15^ and Ethiopia^12,13^ in 2005; Botswana^20,28^ and Lesotho^21^ in 2008; Zimbabwe^30^ in 2010; and Ghana^25^ and Namibia^19^ in 2012. These plans called for laboratory strengthening and development of QMS, some specifying accreditation goals. For example, the laboratory strategic plan in Botswana called for implementation of QMS in all laboratories by 2014 and accreditation of four district-level laboratories by 2013 and two national-level laboratories by 2014.^20^ In Kenya, the Ministry of Health set a goal to accredit all national and regional level public laboratories^10,14^ and established a National Accreditation Steering Committee to coordinate accreditation activities. The Rwanda Ministry of Health aimed to enroll all 48 central and district hospital laboratories in the accreditation preparation process,^29^ and the Ministry of Health in Mozambique established a National Laboratory Policy, which outlined their commitment to implement QMS and to pursue accreditation for reference and provincial hospital laboratories.^18^ The focus on laboratory quality improvement led these countries to implement the SLMTA programme in order to help them improve their quality management.

### Implementation

The standard SLMTA implementation model includes three workshops followed by periods of several months for laboratories to implement improvement projects, usually with onsite support and mentorship.^1^ The SLMTA programme is evaluated through audits based on the SLIPTA checklist.^1^

Several countries have customised SLMTA implementation to meet their needs. In Cameroon, SLMTA workshops were decentralised and conducted on site, allowing many more staff to participate in training.^1,22,26^ The SLMTA team in Vietnam developed an electronic tool for SLIPTA audits so as to improve timeliness and accuracy of audit results and reduce language barriers.^23^ In Rwanda, performance-based financing was used in one cohort, in which payment was provided to laboratories based on SLIPTA audit scores to incentivise continuous quality improvement.^29^ In several programmes, non-laboratory personnel participated in the SLMTA training, including hospital managers and administrators^9,22,26^ as well as clinicians.^13,22,29^ Several countries have established departments or workgroups dedicated to the implementation of quality management. For example, Zimbabwe’s National Quality Assurance Program established a Training and Mentorship Department;^30^ Kenya’s Ministry of Health created a National Accreditation Steering Committee to coordinate laboratory accreditation activities;^14^ and Mozambique established a National Laboratory Technical Working Group to build a framework for a national laboratory quality improvement programme, to lead and coordinate its implementation and to monitor and maintain results.^18^

Individual facilities also established quality improvement programmes. For example, a hospital in Cameroon developed a Quality Improvement Task Force to coordinate quality improvement efforts.^9^ One laboratory in Kenya formed a tiered accreditation team structure, including a Management Team, Quality Assurance Team and Section Teams, to run the programme and lead the laboratory to international accreditation.^10^

### Capacity building

With the rapid expansion of the SLMTA programme, the need to build capacity for more SLMTA trainers, mentors and auditors has been identified.^1,16^ Maruta et al. summarise global efforts to develop trainers and master trainers using a training-of-trainers (TOT) strategy with teach-back methodology.^16^ The TOT course is an intensive two-week training course taught by master trainers, in which candidate trainers learn to teach the 44 activities in the SLMTA curriculum (through a combination of skills learning, practice and feedback) and to follow set guidelines for programme implementation. As of the end of 2013, 433 trainers and 38 master trainers have been produced, and the vast majority (97% and 87%, respectively) are based in developing countries. TOT courses have been held in Botswana, Dominican Republic, Ethiopia, Ghana, Kenya, Mozambique, Nigeria, Rwanda, South Africa, Tanzania, Vietnam and Zimbabwe and have been conducted in English, Portuguese, Spanish and Vietnamese.

Critical considerations include ensuring that TOT graduates are utilised effectively and that fidelity of implementation is maintained as the programme expands. The Maruta et al. study found a 92% utilization rate of TOT graduates, with 97% of participants reporting that the TOT trained them either well or extremely well for implementing SLMTA.^16^ Furthermore, global data suggest that training quality has been maintained, as the 132 laboratories that implemented SLMTA during the first year (2010) had the same mean improvement (24 percentage points) as the 170 laboratories that implemented SLMTA in subsequent years (2011–2013).^33^

The development of mentors and auditors has not been summarised globally, although several studies report country-specific efforts. In Cameroon, 11 mentors and seven auditors have been trained to support programme scale-up.^26^ In Rwanda, 17 local mentors were trained to roll out the SLMTA programme.^29^ In Mozambique, 15 auditors were trained^18^ and in Ghana, 15 mentors and 11 auditors were trained.^25^ Several papers discussed national plans to train additional mentors and auditors.^8,18,19,25,26,29^ One critical consideration for auditor training is to ensure high qualifications and consistency between them. Several studies discussed the variability of auditor expertise and reliability as a serious limitation of both the programme and their reported results.^8,29,33^

### Partnership

The rapid and widespread expansion of the SLMTA programme could not have occurred without the active participation of an extensive network of partners. These various partners have been instrumental in all aspects of programme development and implementation (Table 2). Primarily funded through PEPFAR^1^ and developed under the leadership of the US Centers for Disease Control and Prevention (CDC), Ministries of Health have implemented the programme with support from international organisations, institutions and private companies, parastatal organisations and local non-governmental organisations. One study focused on the use of partnership to implement SLMTA in 15 laboratories in Ghana, concluding that ‘building in-country capacity through local partners is a sustainable model for improving service quality in resource-constrained countries’ and that ‘local partners, when supported and managed adequately, can achieve great results at a reasonable cost’.^25^

**TABLE 2:** Partners contributing to the SLMTA programme as reported by published studies.

Type of Organisation	Acronym	Name of organisation	Programme component												
			Laboratory management framework development	SLMTA development	Funding	Implementation	Coordination, logistical support and technical assistance	SLMTA training	Mentorship and supervisory visits	Auditing	Additional training: Auditing	Additional training: Mentorship	Additional training: Training-of-Trainers	Additional training: Good clinical laboratory practice	Additionaltraining: ISO 15189 standards
**Government**	**CDC**	**US Centers for Disease Control and Prevention**	-	1	20, 26	8, 14, 22	10, 11, 17, 21, 25, 28, 30	7, 18, 22	10, 18	18	-	-	12	-	-
	**PEPFAR**	**US President’s Emergency Plan for AIDS Relief**	-	-	1, 8, 19, 10, 14, 15, 17, 20, 25, 31	-	-	-	-	-	-	-	-	-	-
**International**	**ACILT**	**African Centre for Integrated Laboratory Training**	-	-	-	-	-	-	-	-	-	-	12	-	-
	**AFENET**	**African Field Epidemiology Network**	-	-	-	14	11	-	-	-	-	-	-	-	-
	**APHL**	**Association of Public Health Laboratories**	32	-	-	-	21	21	-	-	-	-	-	-	-
	**ASCP**	**American Society for Clinical Pathology**	-	1	-	14	-	7, 12		29	-	-	-	-	-
	**ASLM**	**African Society for Laboratory Medicine**		-	-	-	-	-	-	7, 18, 26, 29	-	-	-	-	-
	**ASM**	**American Society for Microbiology**	32	-	-	-	-	-	8	-	-	-	-	-	-
	**CHAI**	**Clinton Health Access Initiative**		1	-	-	21	21	22	-	-	22, 26	-	-	-
	**CLSI**	**Clinical and Laboratory Standards Institute**	32	-	-	-	-	-	-	-	25	-	-	-	-
	**MSH**	**Management Sciences for Health**	-	-	-	14	-	-	-	-	-	-	-	-	10
	**WB-EAPHLN**	**World Bank’s East Africa Public Health Laboratory Network Project**	-	-	29*	14	-	-	-	-	-	-	-	-	-
	**WHO AFRO**	**World Health Organization’s Regional Office for Africa**	-	1	-	-	-	-	-	-	-	-	-	-	-
**Institutional**	**AMPATH**	**Moi University, School of Medicine Academic Modelling Providing Access to Healthcare Laboratory, Eldoret**	-	-	-	-	-	-	15	-	-	-	-	-	-
	**ICAP**	**Columbia University’s International Center for AIDS Care and Treatment Programs**	-	-	-	-	-	-	12	-	-	-	-	-	-
	**ITECH**	**University of Washington’s International Training and Education Center on HIV**	-	-	-	-	-	-	12	-	-	-	-	-	-
	**JHU-TSEHAI**	**Johns Hopkins University Technical Support for Ethiopian HIV/AIDS Antiretroviral Treatment Initiative**	-	-	-	-	-	-	12	-	-	-	-	-	-
	**KAVI**	**Kenya AIDS Vaccine Initiative**	-	-	-	14	-	-	-	-	-	-	-	-	-
	**KEMRI**	**Kenya Medical Research Institute HIV-Research Laboratory, Kisumu**	-	-	-	-	-	-	15	-	-	-	-	-	-
	**UCSD**	**University of California San Diego**	-	-	-	-	-	-	12	-	-	-	-	-	-
	**WRP**	**Walter Reed Program Research Laboratory, Kericho**	-	-	-	-	-	-	15	-	-	-	-	-	-
**Private**	**BD**	**Becton Dickinson**	32	-	-	-	-	-	-	-	-	-	-	-	-
**Parastatal**	**BOBS**	**Botswana Bureau of Standards**	-	-	-	-	-	-	20, 28	20	-	-	-	-	-
	**GSA**	**Ghana Standards Authority**	-	-	-	-	-	-	-	-	25	-	-	-	25
	**KENAS**	**Kenya Accreditation Service**	-	-	-	-	-	-	-	10, 14, 17	-	-	-	-	-
	**SANAS**	**South African National Accreditation System**	-	-	-	-	-	-	-	7, 10	10, 22	-	-	22	-
**NGO**	**AGHPF**	**A Global Health Care Public Foundation**	-	-	17**	14	10	17	17	-	-	-	-	-	14
**Local NGO**	**APIN**	**AIDS Prevention Initiative in Nigeria**	-	-	-	8	-	-	-	-	-	-	-	-	-
	**GHSS**	**Global Health Systems Solutions**	-	-	-	22, 26	-	25	9, 26	-	-	25	-	-	-
	**ZINQAP**	**Zimbabwe National Quality Assurance Program**	-	-	-	30, 31	-	-	-	-	-	-	-	-	-

SLMTA, Strengthening Laboratory Management Toward Accreditation; NGO, non-governmental organization; ISO, International Organization for Standardization.

*Performance-based financing; **Facility and equipment upgrades.

Numbers in table correspond to reference numbers of published studies.

### On-site support and mentorship

On-site support and mentorship are key components of the SLMTA programme, as mentors are expected to provide in-depth support after workshops to assist laboratory personnel in implementing changes.^32^ Support and mentorship models have ranged from no site visits or mentorship,^8,20^ occasional visits,^14^ periodic visits of several days to several weeks,^8,10,16,28,29,30^ to embedded mentors working full time in their assigned laboratories for extended periods^11,14,18,22,25,26,29,30^ (Table 3). Kenya has piloted a novel mentorship model, ‘twinning’ public laboratories with local state-of-the-art research laboratories. This institutional mentorship approach holds promise not only for laboratory improvement, but also for fostering long-standing sustainable partnerships between public health and research laboratories.^15^

**TABLE 3:** Mentorship models reported by SLMTA studies.

Study	Comparison	Selection	Results	Conclusion
Audu et al.^8^	One mentored laboratory (four visits of 2–4 weeks each) versus one non-mentored laboratory.	Purposive, based on specialty of the laboratory and expert availability from partner agency.	Mentored laboratory increased from 66%at baseline to 95%at exit (29 percentage points). Non-mentored laboratory increased from 80%at baseline to 93%at exit audit (13 percentage points).	‘The laboratory with expert on-site mentorship improved farther and steadier, achieving a score of five stars. Our results suggest that laboratories should consider using on-site mentorship in order to augment the impact of SLMTA in implementing quality improvement.’
Hiwotu et al.^12^	The 23 laboratories in Cohort 1 had more extensive supportive supervision (68 hours per laboratory) versus the 21 laboratories in Cohort 2 (two hours per laboratory).	Purposive, based on timing due to partner support for Cohort 1.	Extra support laboratories increased from 40%at baseline to 58%at exit audit (18 percentage points). Limited support laboratories increased from 42%at baseline to 53%at exit audit (11 percentage points).	‘Our data suggest that supportive site visits were critical with regard to reinforcing the knowledge and motivation offered during the trainingin order to achieve the expected behavioural changes required for quality improvement.’
Makokha et al.^15^	Three laboratories paired with research laboratories (institutional mentorship) versus five laboratories receiving standard mentorship (once per month for five days).	Purposive, based on proximity to research laboratories.	Twinned laboratories increased from 36%at baseline to 80%at exit audit (44 percentage points). Non-twinned (standard mentorship) laboratories increased from 30%at baseline to 68%at exit audit (38 percentage points).	‘The partnership used by the twinning model holds promise for future collaborations between ministries of health and state-of-the-art research laboratories in their regions for laboratory quality improvement.’
Mokobela et al.^20^	Three laboratories received mentorship from the Botswana Bureau of Standards (BOBS) versus four laboratories with no mentorship.	Purposive, laboratories recently relocated to new facilities and were designated as Centres of Excellence in medical specialties.	Mentored laboratories increased from 53%at baseline to 74%at exit audit (21 percentage points). Non-mentored laboratories increased from 49%at baseline to 57%at exit audit (8 percentage points).	‘Supplemental mentorship and training may have contributed to the success amongst BOBS-mentored laboratories, which showed greater improvements in SLIPTA audit results. However, it is important to note that the small number of laboratories and lack of random assignment to BOBS mentorship limits the ability to draw definitive conclusions regarding this comparison.’
Nzabahimana et al.^29^	Cohorts I and III received standard mentorship (five days after each workshop). Cohort II received standard mentorship plus embedded mentorship for two weeks per month for eight months.	Purposive, based on timing.	Embedded mentorship laboratories (Cohort II) increased from 28%at baseline to 70%at exit audit (42 percentage points). Standard mentorship laboratories in Cohorts I and III increased from 43%and 32%at baseline to 73%and 56%at exit audit (30 and 34 percentage points, respectively).	‘Performance-based financing, intensive mentoring and supplementary financial resources may have contributed to gains in Cohort II laboratories.’
Nzombe et al.^30^	Four mentorship models: (1) laboratory manager mentorship after SLMTA (four laboratories), (2) one week per month mentorship after SLMTA (four laboratories), (3) cyclical embedded mentorship after SLMTA (three laboratories), (4) cyclical embedded mentorship incorporated with SLMTA (eight laboratories).	Purposive, based on location, funds, resources, staff allocation, and timing	Median improvements were 17 percentage points for Model 1, 23 percentage points for Model 2, 25 percentage points for Model 3. Model 4 laboratories increased 39 percentage points from pre-SLMTA baseline to exit audit.	‘The addition of mentorship had a beneficial effect on the laboratories over and above the effect of SLMTA training alone… We were not able to conclude that one model was better than the others… Countries should carefully consider which mentorship model or models would be best suited to their individual situation.’

SLMTA, Strengthening Laboratory Management Toward Accreditation.

Well-planned scientific studies are lacking with regard to the effectiveness of mentorship overall or the relative effectiveness of various models. All of the SLMTA-related studies conducted to date have serious methodological flaws, primarily the lack of random assignment to mentorship models, lack of control groups and small sample sizes. Despite these limitations, several post-hoc analyses comparing results in mentored laboratories to those in non-mentored laboratories,^8,20^ as well as intensive mentorship to less intensive mentorship,^15,29^ concur that mentorship appears to be beneficial (Table 3). These findings agree with an earlier study that found that SLIPTA scores increased in four Lesotho laboratories after mentorship, although no control laboratories were used on which to base a comparison.^42^ Despite the lack of solid scientific evidence, there is a general belief that mentorship is an important component of the SLMTA programme and a critical factor for success.

### Success factors

Numerous factors have been identified by authors as being critical to the success of SLMTA implementation. At the facility level, many authors reported the importance of engaging hospital and senior management from the beginning so as to ensure their buy-in and ownership of the programme^7,12,17,20,25,26,28,29,30^ and to promote institutionalisation and thus sustainability.^18^ Many also noted the importance of a strong commitment and team spirit amongst the laboratory staff^7,9,10,18,20,28,29^ and a willingness to build a culture of quality^17,26^ and problem solving.^20^ The various components of the SLMTA programme – including the how-to guidance provided by the workshops;^7,10,17,28,32^ mentorship^7,10,11,25,30^ and supervisory visits^7,12,18,21,32^ to keep laboratories on track; improvement projects;^20,26,32^ and ensuring that both staff^11,17,32^ and leadership^17^ are accountable and motivated – were viewed as critical. In addition, authors noted that it is essential to measure what has been accomplished through audits using the SLIPTA checklist^10,11,17,18,21,32^ and analysis of other key indicators.^28^ These data are powerful means to recognise and motivate staff, determine further actions needed^17^ and provide information as an advocacy tool.^16^ Finally, ongoing communication with hospital management^21^ and clinical staff^9,17,26,28^ is critical so as to ensure continued focus on patient care and support for future activities.

At the country level, it is critical to ensure clear commitment and ownership within the Ministry of Health in order to improve laboratory quality at all levels, including development of a national laboratory policy and strategic plan, establishment of a laboratory technical working group and provision of financial support.^1,21,25^ Early engagement of key stakeholders and partners,^11,21,25^ followed by effective communication and continuous advocacy for laboratory quality,^9,12,18^ were identified as important components of success, as were the development of a programme implementation plan^9,10,11^ that includes human resource needs for trainers, auditors and mentors;^1^ a plan to ensure sufficient geographic coverage through careful site selection;^1,18^ and establishment of specific programme goals.^18^

### Challenges

Challenges at both the facility and programme levels were identified, as well as those beyond the scope of the programme that affect the programme. At the facility level, common concerns surrounded the difficulty of engaging non-SLMTA-trained laboratory staff,^1,10,12,13^ as well as hospital management and regional health bureaus^1,12,13^ and ensuring harmonisation with other hospital improvement programmes.^12^ It was noted that behavioural change requires time, commitment and consistent support.^32^ Also documented were difficulties in: providing sufficient site support;^1,12^ ensuring that staff understand the requirements of QMS^8^ and the International Organization for Standardization (ISO) 15189 standard;^8,10,12^ how to conduct internal audits;^25^ and the importance of establishing quality in laboratory testing.^22^ Implementing a QMS is a difficult process; particularly noted were the challenges of: balancing the requirements of multiple functions within a laboratory;^20^ establishing root causes of nonconformities;^8,14^ equipment maintenance and outages;^13,22^ development of method validation;^10^ space shortages;^10^ document review and maintenance;^7,20,26^ insufficient time to implement improvement projects;^21^ and the general concern that the entire process required more time and resources than anticipated.^13^

At the programme level, the shortage of well-trained mentors^11,13,15,25^ was a common concern, as were the lack of trainers^22^ and auditors.^23^ In addition, it was noted that mentors and other implementers have competing duties, since they are generally not dedicated solely to SLMTA activities,^18,30^ which may lead to suboptimal utilisation rates of trainees.^16^ Furthermore, language and communication barriers amongst mentors, trainers and auditors can be a challenge,^23,25^ exacerbating the shortages.

Also reported were higher-level challenges that had an impact on programme implementation. The most-commonly reported challenge was that of staff attrition.^10,11,13,17,20,21,29,31,32^ One study of programme implementation in the Caribbean region reported the following:

[*E*]nsuring a sufficient number of well-qualified laboratory workers is an ongoing challenge, exacerbated by high levels of attrition as staff that have benefitted from government-supported training leave the public sector for more lucrative jobs in the private sector, either locally or overseas. Thus the remaining staff are overworked, reducing the amount of time available for training and quality improvement activities.^11^

In a study from Rwanda, the only laboratory whose scores decreased from baseline to exit audit lost both their quality officer and laboratory manager during the programme;^29^ and a laboratory in Kenya found that patient complaints increased as a result of high staff turnover.^17^ Shumba et al. suggest several strategies to reduce staff attrition, including encouraging Ministries of Health and supervisors to agree not to reassign trained staff for a period of time, providing financial incentives to participants at the completion of SLMTA and using binding contracts in which participants agree to remain on the job for a specified period.^31^ Decentralised training may also help reduce the effect of attrition; in Cameroon, the authors concluded that, ‘[i]n the decentralised model, the majority of laboratory staff are trained to implement QMS, reducing the impact of attrition of a few trained staff members’.^22^ In addition, studies reported a general shortage of qualified laboratory staff, especially staff with QMS expertise.^11,12,22,25,29^ Also noted was a lack of quality manuals, guidelines and procedures^12^ to provide clear direction, as well as a lack of national strategic plans^22^ to define stakeholders and facilitate coordination with partners.^13^ These issues, when coupled with institutional bottlenecks,^22,25^ slow procurement processes^10^ and lack of or limited accreditation preparation budgets,^12,25^ further hamper improvement efforts. Finally, the existing low level of quality management in developing countries^33^ suggests that much work is needed to ensure sufficient quality of laboratory services to provide for public and personal health needs.

### Limitations to the study

This review is subject to several limitations. Primarily, the results may not be representative of the programme as a whole, or a comprehensive account of all laboratories’ experiences. For example, whilst these studies mentioned some 30 partners who helped to develop or implement the SLMTA programme, others were undoubtedly missed.

### Feedback on the SLMTA programme

Overall, the published literature was strongly in favour of the SLMTA programme, with investigators and public health officials reporting satisfaction with the results. Nkengasong and Birx suggest that ‘with the introduction of SLMTA, the prospects of implementing sustainable quality-assured laboratory medicine seem to be a reality in developing countries’.^24^ Other investigators agreed, saying that SLMTA ‘was found to be a practical option that yielded positive results for strengthening laboratories’^20^ and also that ‘[t]he tremendous improvement… shows that SLMTA coupled with mentorship is an effective, user-friendly, flexible and customisable approach to implementation of laboratory QMS’.^11^

A study of attitudes of health professionals in Ethiopia found that laboratory professionals had a supportive perception of SLMTA, whilst some hospital chief executive officers ‘were more sceptical of SLMTA and raised concerns regarding programme costs and the prolonged process associated with implementation’.^13^ A hospital director in Cameroon disagreed, saying that:

SLMTA is an invaluable tool for every lab director, every hospital manager and health policy maker because of its value in ensuring quality improvement within the laboratory and its potential in contributing to strengthening the entire health system at little or no cost.^9^

## Future directions

**FIGURE 1:**
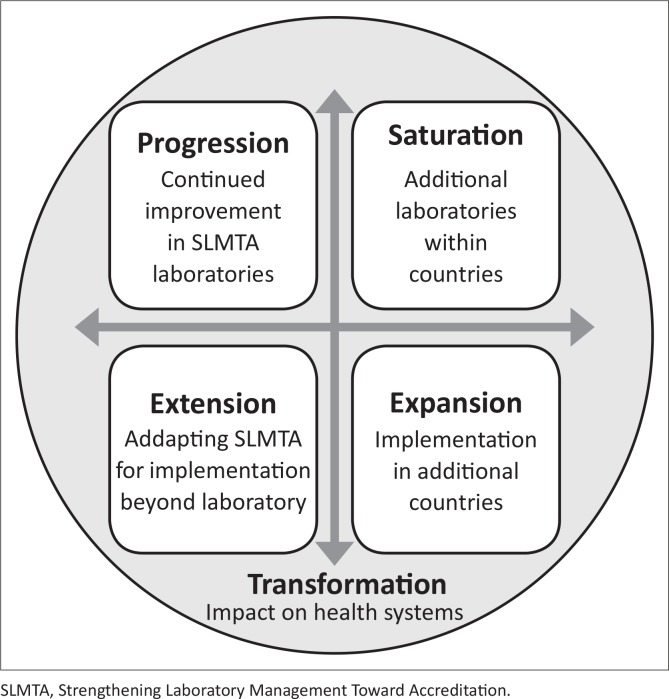
Future directions of the SLMTA programme.

The SLMTA programme is expanding rapidly and authors have identified an urgent need to sustain the gains and move forward in four strategic directions to lead to transformation of overall health systems and patient care (Figure 1).

### Progression (continued improvement in SLMTA laboratories)

Several authors have discussed the need to ensure that laboratories sustain gains made and continue to move forward.^8,11,17,20,26,27,29^ Yao et al. point out that quality improvement should be seen as an ongoing journey and that SLMTA provides the tools needed to ensure better patient care;^1^ Ntshambiwa et al. concur that SLMTA has ‘helped lay a firm foundation for further advancements in patient care’.^28^ Rwanda’s first two SLMTA cohorts not only sustained their results at a surveillance audit a year after SLMTA completion, but increased their scores by a median of 10 percentage points.^29^ Globally, 92 laboratories have completed surveillance audits; 62% further increased their score.^33^ Nkrumah contends that indigenous capacity building is critical in order to ensure sustainability,^25^ whilst Maruti et al. focus on the need to change the laboratory culture by establishing universal rules, teaching staff the principles and techniques of quality improvement, continually reinforcing the behaviours by integrating them into daily routines and engaging hospital stakeholders.^17^ Audu et al. argue that ‘sustainability is a common concern for any improvement programme; once the intense focus of implementation ceases, special efforts and continued supervision are required so as to ensure that old habits do not return’.^8^ Maina et al. also focus on post-SLMTA sustainability, identifying internal audits and corrective action as catalysts for continued improvement.^14^ Noble et al. commend the achievements made by laboratories to date, cautioning:

But to them we put forward this challenge: whilst it is a great achievement to reach a level of success where the requirements of accreditation are met, the true accomplishment is reaching the point where the level of quality is an everyday practice and expectation, and the laboratory is ‘accreditation-ready’ over and over. When the inevitable slips and mistakes occur in laboratories that are accreditation-ready, the processes of error detection, correction and improvement, and progress back to quality, must occur quickly, smoothly and sustainably.^27^

As the SLMTA programme matures, studies measuring the long-term sustainability of results and examining factors associated with continued progress will be critical for ensuring the enduring impact of the programme.

### Saturation (additional laboratories within countries that have implemented SLMTA)

Several countries have established formal plans for country-wide QMS implementation, using the SLMTA programme as the central improvement tool.^18,25,29^ Many have already implemented second (13 countries), third (six countries) and further cohorts (three countries) as they expand SLMTA nationally; of the 21 countries that began SLMTA in 2010–2011, 16 (76%) have thus far implemented additional cohorts.^33^ Kenya has conducted six rounds of SLMTA, whilst Lesotho has reached near saturation, with SLMTA implemented in 18 of its 19 laboratories.^33^

Several authors discuss the next steps toward achieving greater saturation within countries. Most common amongst these is the need to develop additional local trainers, mentors and auditors,^1,11,13,16,22,23,25,30^ as well as educating the general workforce through pre-service training.^24^ Guevara et al. suggest that ‘there is now a need to internalise the programme and transition it to local governments and other donors in order to facilitate expansion and ensure sustainability’,^11^ whilst Lulie et al. argue that further efforts are needed to ‘decentralise responsibility from the government to the management at their facilities’.^13^

It is evident that not all laboratories in a country’s health system need to be accredited; however, all laboratories must maintain a culture of continuous quality improvement. Nkengasong and Birx discuss the need for countries to identify a ‘tipping point’ or threshold of laboratories that must be accredited in order to establish this culture and ‘increase confidence in quality-assured laboratory medicine for evidence-based patient management’.^24^ Once accreditation goals are defined in national laboratory strategic plans, a SLMTA implementation plan can be developed with clear priorities to help guide laboratories in the tiered health system to achieve their goals. Masamha et al. suggest that, in addition to accreditation, countries could track the progress of quality systems with indicators such as:

[*the*] number of provinces with dedicated quality management officers; percentage of laboratories audited in the previous 12 months; percentage of audited laboratories demonstrating improvement as measured by the SLIPTA checklist; and percentage of laboratories implementing external proficiency testing for select services.^18^

### Expansion (implementation in additional countries)

SLMTA implementation started with 11 countries in 2010 and spread to an additional 10 in 2011, 15 in 2012 and 11 in 2013.^33^ Because PEPFAR is the primary funding source, SLMTA to date has been rolled out largely in PEPFAR-supported countries^43^ (43 of the 47 countries implementing SLMTA are PEPFAR-funded, 91%). As of the end of 2013, 75% of the 57 PEPFAR-supported countries have implemented SLMTA, with most of the remaining countries located in Asia.

To date, SLMTA has been implemented in 38% of low-income countries and 26% of lower-middle-income countries, based on World Bank classifications.^44^ Further expansion beyond PEPFAR-supported countries to other low- and lower-middle-income countries will require the identification of additional global partners to provide funding and implementation support.

### Extension (adapting SLMTA for implementation beyond the laboratory)

To take full advantage of the benefits of improved laboratory quality, improvements will need to be made outside the scope of the laboratory as well. In her commentary, the laboratory director for Namibia’s Ministry of Health and Social Services (MoHSS) explains:

As quality improvements become institutionalised in hospital laboratories, it is becoming evident that entire hospital systems are in dire need of similar quality improvement programmes. The Namibia MoHSS calls on international agencies to develop and adapt programmes such as SLIPTA and SLMTA for use throughout hospital systems so as to ensure continuous quality patient care.^19^

Along the same lines, Eno et al. report on the experience of one hospital that adapted the SLMTA tool for wider implementation, inspired by successful implementation of the programme in their laboratory.^9^ Results were encouraging, with ‘steady improvement in service delivery’; reduced patient wait times, infection rates and stillbirths; and increased hospital revenue. The authors concluded that ‘[s]uch a programme has the potential to impact positively on hospital quality systems; consideration should be made for development of a formal SLMTA-like programme for hospital quality improvement … expanding the Strengthening *Laboratory* Management Toward Accreditation programme into one for Strengthening *Hospital* Management Toward Accreditation’.^9^

### Transformation (impact on health systems and patient care)

As SLMTA continues to grow, it has the potential to have a profound and lasting impact on health systems and patient care. In a report on the impact of PEPFAR, the Institute of Medicine concluded that:

PEPFAR’s laboratory efforts have had a fundamental and substantial impact on laboratory capacity in countries. This laboratory infrastructure and capacity has been, and can continue to be, leveraged to improve the functioning of countries’ entire health systems.^36^

There is a growing movement toward establishing a culture of quality at all levels of service in order to care for patients,^24^ including not only the laboratory but the pharmacy, clinics, maternity, surgical rooms and others. As quality improves, there is a need to measure the impact on patient outcomes through well-defined and rigorous programme evaluation. Such data will provide local, national and global decision makers with the evidence needed to justify expenditures and implement the most appropriate solutions for their given situations.
